# Preparation and Characterization of Immobilized Lipase from Pseudomonas Cepacia onto Magnetic Cellulose Nanocrystals

**DOI:** 10.1038/srep20420

**Published:** 2016-02-04

**Authors:** Shi-Lin Cao, Yu-Mei Huang, Xue-Hui Li, Pei Xu, Hong Wu, Ning Li, Wen-Yong Lou, Min-Hua Zong

**Affiliations:** 1 State Key Laboratory of Pulp and Paper Engineering, South China University of Technology, Guangzhou 510640, China; 2 School of Chemistry and Chemical Engineering, South China University of Technology, Guangzhou 510640, China; 3 Lab of Applied Biocatalysis, School of Food Science and Engineering, South China University of Technology, Guangzhou 510640, China

## Abstract

Magnetic cellulose nanocrystals (MCNCs) were prepared and used as an enzyme support for immobilization of Pseudomonas cepacialipase (PCL). PCL was successfully immobilized onto MCNCs (PCL@MCNC) by a precipitation-cross-linking method. The resulting PCL@MCNC with a nanoscale size had high enzyme loading (82.2 mg enzyme/g) and activity recovery (95.9%). Compared with free PCL, PCL@MCNC exhibited significantly enhanced stability and solvent tolerance, due to the increase of enzyme structure rigidity. The observable optimum pH and temperature for PCL@MCNC were higher than those of free PCL. PCL@MCNC manifested relatively higher enzyme-substrate affinity and catalytic efficiency. Moreover, PCL@MCNC was capable of effectively catalyzing asymmetric hydrolysis of ketoprofenethyl ester with high yield of 43.4% and product e.e. of 83.5%. Besides, immobilization allowed PCL@MCNC reuse for at least 6 consecutive cycles retaining over 66% of its initial activity. PCL@MCNC was readily recycled by magnetic forces. Remarkably, the as-prepared nanobiocatalyst PCL@MCNC is promising for biocatalysis.

Enzymes are widely used in food, medicine, energy and other industrial fields due to their high catalytic efficiency and mild green reaction conditions^1^,^2^. However, further industrial application of free enzymes is restricted due to a number of disadvantages such as high cost, poor operational stability, and difficulties in recovery and reuse^3^,^4^. Immobilization of enzymes can effectively solve these obstacles. There are a number of new technologies and methods in the field of enzyme immobilization^5^, however, efficient and simple immobilization methods and tools require further investigation.

Cellulose nanocrystals (CNCs) have attracted increased attention due to their high surface-to-volume ratio, high aspect ratio, and high biocompatibility^6^. These excellent physicochemical characteristics of CNCs can enhance the activity and stability of glucose oxidase^7^, peroxidase^8^, papain^9^ and lysozyme^10^. However, the CNCs are difficult to recycle from the reaction system due to highly stable dispersion, thus limiting their applications.

Recently, our group reported a novel low-cost magnetic CNC (MCNC) nanomaterials using a simple co-precipitation-electrostatic-self-assembly technique^11^. This MCNC nanomaterial had satisfactory biocompatibility. Moreover, this MCNC carrier can easily be separated under a magnetic field. However, using this novel nanomaterial as an enzyme carrier for lipase immobilization requires further study. Lipases (EC 3.1.1.3), due to their advantages including high selectivity and wide substrate specificity^12^, have been widely used in food stuffs, biodiesel production, cosmetics and pharmaceuticals^13^,^14^,^15^. Hence, we selected a lipase from *Pseudomonas cepacia* (PCL) as our model enzyme for immobilization. It is of interest to study whether controlled deposition of free enzyme can occur on the MCNC surface with precipitant and subsequent cross-linking with crosslinking agent (named the precipitation-cross-linking process).

Thus, in the present study, MCNC was prepared, and PCL was successfully immobilized onto MCNC via the precipitation-crosslinking method. Furthermore, a comparative study of PCL@MCNC and free PCL was performed, and the results showed that the PCL@MCNC had better catalytic efficiency and stability than free PCL.

## Results

### Characterization of MCNC and **PCL@MCNC**

**Figure 1:**
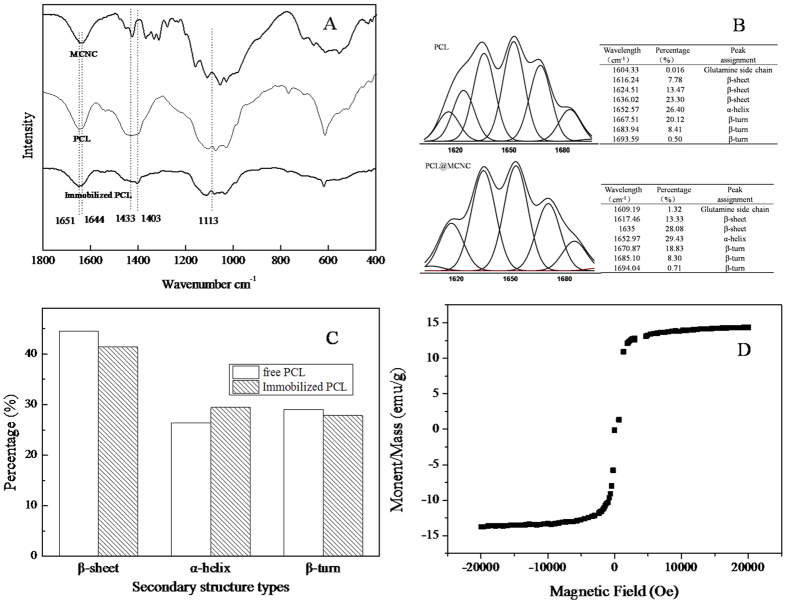
Secondary structures analysis of PCL and PCL@MCNC via FT-IR. FT-IR spectra for MCNCs, PCL,PCL@MCNC (**A**); Amide I fitting results of PCL,PCL@MCNC (**B**); Secondary structures changes of PCL and PCL@MCNC (**C**); Hysteresis loops of MCNC (**D**).

The FT-IR spectra of MCNC, free PCL and PCL@MCNC are shown in Fig. 1A, respectively. In Fig. 1, as shown from the spectra of PCL@MCNC, the bands at 1433 cm^−1^ became weaker (1403 cm^−1^), which was probably caused by the electrostatic interactions between MCNCs and free enzyme. The vibrational frequency at 1113 cm^−1^ which was a typical frequency of MCNCs caused by the asymmetrical ring^16^ was seen in the spectra of PCL@MCNC, and this indicated the successful attachment of PCL and MCNCs. By comparing the spectrum of free PCL with PCL@MCNC (Fig. 1A), the spectra showed similar bands for amide I and II. Nevertheless, the characteristic peak of free PCL attributed to intermolecular bonding in the protein at 1651 cm^−1^ shifted to 1650 cm^−1^, which originated from a strong hydrogen bond with the peak of (CONH_2_) in MCNCs at 1644 cm^−1^, indicating that the PCL was successfully linked to the MCNCs support, as described previously^17^,^18^. Therefore, from the FT-IR results shown in Fig. 1A, the connection between MCNCs and PCL@MCNC was successfully established using the cross-linker glutaraldehyde.

Previous studies indicated that the second derivative FT-IR spectra in the amide I region of an enzyme was used as a particularly sensitive probe of protein conformation^19^,^20^,^21^. To date, there have also been some reports on the use of the second derivative FT-IR spectra to study the conformational changes of an enzyme after immobilization^22^,^23^,^24^,^25^. Therefore, it was of great interest to comparatively investigate the secondary structure contents of PCL and PCL@MCNC and their conformational changes using FT-IR spectra to get some insight of the stabilization mechanism of the immobilized enzyme PCL@MCNC. Figure 1B,C depicted the amide I fitting results (1700 cm^−1^ − 1600 cm^−1^) and the corresponding secondary structure changes in PCL and PCL@MCNC, respectively. A comparison of the PCL and PCL@MCNC structures indicated that different degrees of change had taken place after the precipitation-cross-linking process. The percentages of β-sheet and β-turn decreased by 3.14% and 1.19%, respectively, and the content of α-helix increased by 3.03%. Obviously, the increased α-helix content of PCL@MCNC relative to PCL contributed to the stability of the enzyme, which was supported by similar observations^9^,^23^,^26^. Also, this could well explain the improvement in stability and organic solvent resistance observed which is detailed below.

**Figure 2:**
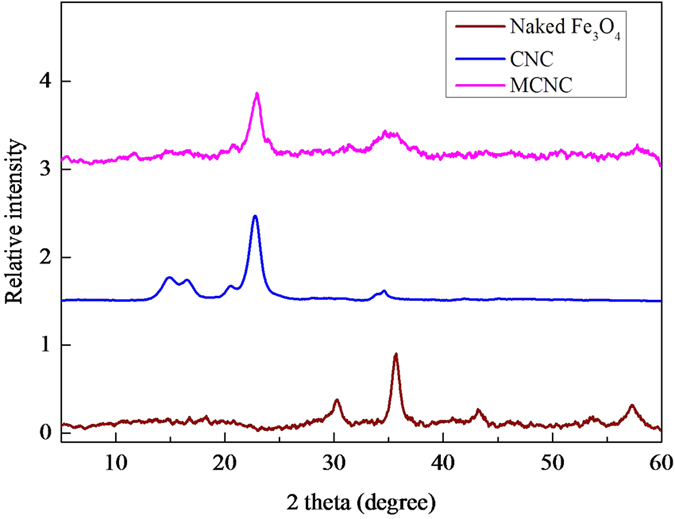
XRD spectra for MCNC, CNC, naked- Fe_3_O_4_.

A vibrating sample magnetometer was used to measure the magnetisation of the as-prepared MCNC at room temperature. As illustrated in Fig. 1D, the magnetic support MCNC had a saturation magnetization of around 14.36 emu/g. A typical XRD pattern of MCNCs and microcrystalline cellulose (MCC) is shown in Fig. 2. The diffraction peaks were 34.3°, 22.8°, 17.1°, and 14.3° which were ascribed to the crystallographic planes of (040), (002), (10−1), and (101)^27^, respectively. These results clearly indicated that the crystalline structure of cellulose was maintained during the MCNC preparation process. In addition, five distinct characteristic diffraction peaks belonging to Fe_3_O_4_ at 2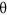 = 30.8°, 36.2°, 44.1°, 53.9° and 57.5° were seen in the MCNCs sample and the two peaks at 30.8° and 44.1° were weak, which confirmed the composition of magnetic Fe_3_O_4_ with the CNC polymer matrix and the weaker intensities of Fe_3_O_4_ after composition, as shown in a previous study^11^,^28^.

**Figure 3:**
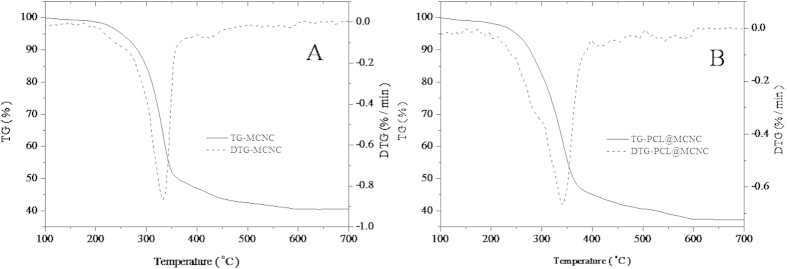
Thermogravimetric (TG) analysis for MCNC(A) and PCL@MCNC.

It was of great interest to characterize the compositions of the as-prepared PCL@MCNC. On the basis of thermogravimetric (TG) analysis for MCNC (Fig. 3A) and PCL@MCNC (Fig. 3B), the mass contents of the main components (PCL, CNC, Fe_3_O_4_) could be estimated. The weight loss of the MCNC sample at 100 to 700 °C was observed at 59.5%, and the content of Fe_3_O_4_ in MCNC was about 40.5% (Fig. 3A). As shown in Fig. 3B, the PCL@MCNC sample lose about 62.7% of its weight at 100 to 700 °C, and the Fe_3_O_4_content in PCL@MCNC was estimated to be 37.3%. The content of CNC in PCL@MCNC could be calculated to be around 54.9% (containing less than 1.6% chitosan). As a consequence, the content of PCL in PCL@MCNC was around 7.8%.

**Figure 4:**
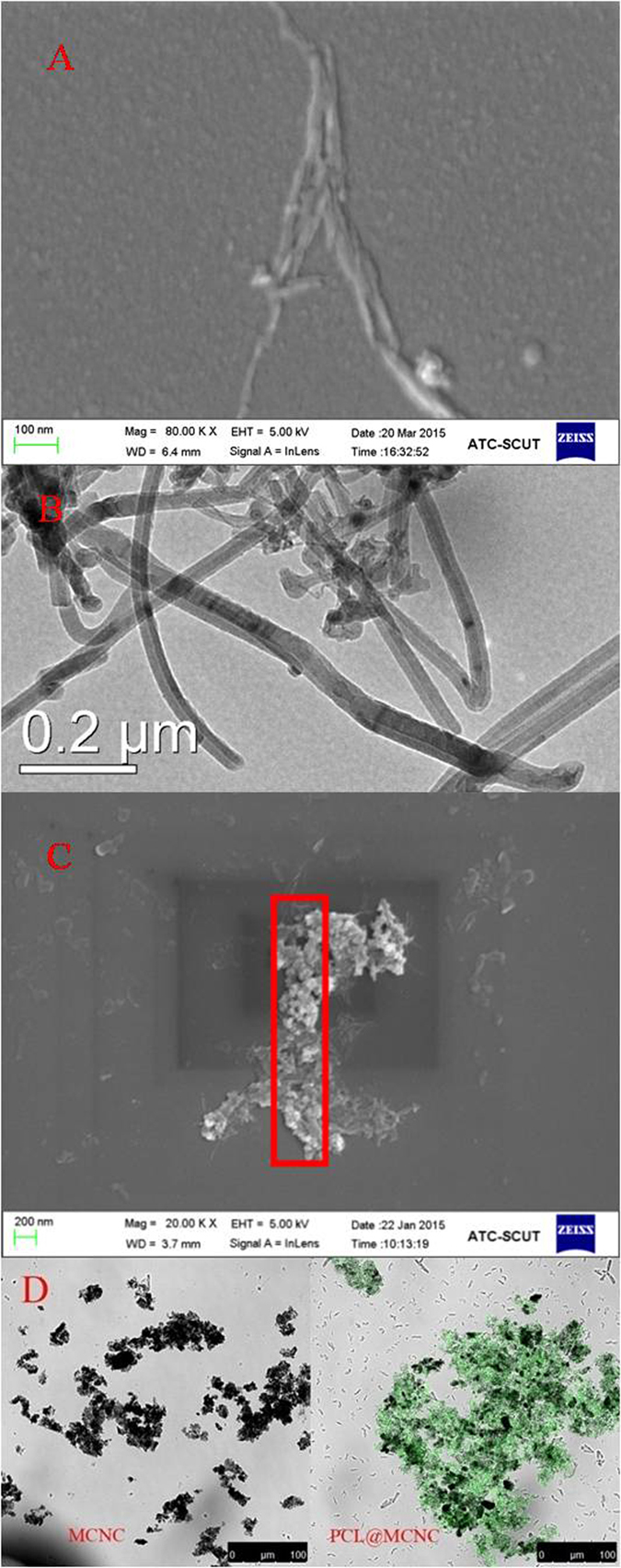
Scanning electron mircophotographs of MCNC (A) and PCL@MCNC (C), transmission electron mircophotographs of MCNC (B); Confocal laser scanning microscopy image of MCNC and PCL@MCNC.

The surface profiles of MCNC and PCL@MCNC were studied using SEM and TEM techniques, respectively, and the observed images were shown in Fig. 4A–C. Figure 3A,B illustrated that the prepared MCNC had the length of approximately 700 nm and the width of around 80 nm, which was very similar to that observed in our previous study^11^. Also, the TEM image showed that the Fe_3_O_4_ particles were loaded on the surface of MCNC and covered by chitosan layer (Fig. 4B). As evident from the SEM image of PCL@MCNC depicted in Fig. 4C, the immobilized enzyme had a rod-like surface morphology. Furthermore, it is noteworthy that the scaffold of the magnetic support MCNC was covered by PCL, suggesting that PCL was successfully loaded onto the surface of MCNC by the precipitation-cross-linking process. To further confirm the presence of the targeted PCL on the prepared MCNC support, we performed the confocal laser scanning microscopy (CLSM) analysis for MCNC and PCL@MCNC. From the CLSM images depicted in Fig. 4D, it was clearly observed that the support MCNC without PCL loading showed no fluorescent signal, while the resulting PCL@MCNC after the immobilization of the fluorescein *iso*-thiocyanate (FITC)-labeled PCL onto the MCNC support displayed strong green fluorescent signal, suggesting the presence of the FITC-labeled PCL on the MCNC support after immobilization.

**Figure 5:**
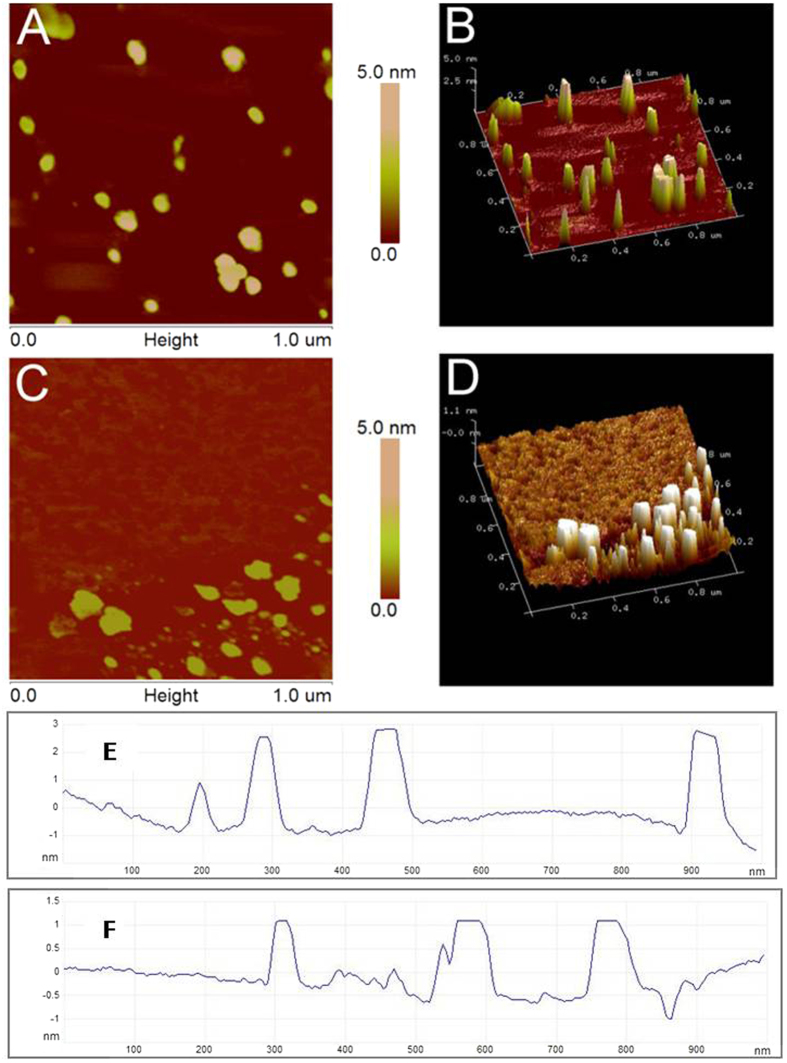
Representative AFM images, three-dimensional figures, and corresponding height profiles of PCL (A,B,E) and PCL@MCNC (C,D,F)

AFM is an effective approach for observing the morphology of PCL and PCL@MCNC. The AFM images clearly showed that the surface morphology of PCL was significantly changed after immobilization onto MCNCs. The mean diameter and height of PCL@MCNC (Fig. 5C,F) were approximately 75.82 nm and 1.7 nm, respectively, while the diameter of free PCL (Fig. 5A,E) was a little smaller (20.84 nm) and the height was greater (2.3 nm), in accordance with a previous report^29^. It is noteworthy that the surface of immobilized PCL (Fig. 5C) showed gaps similar to a network, which demonstrated that there was sufficient cross-linking between the protein molecules. To further characterize the differences in surface topography of PCL, three dimensional AFM images (Fig. 5B,D) were employed to reveal the strong visual impact of the constructional changes. As clearly depicted in Fig. 5B, proteins of the free enzyme were uniformly dispersed with no obvious aggregation. However, after the precipitation-cross-linking process, the proteins (Fig. 5D) began to form irregular aggregates on the surface of MCNC with decreased height and increased width, and subsequently produced a porous mesh structure by integration and cross-linking in longitudinal and transverse directions.

These observations led to the conclusion that the structure of the PCL@MCNC was relatively compact, due to the strong combination of inter- and intramolecular interactions and the network gaps between the molecules exerted an attractive driving force in the catalytic reaction, to a certain extent, probably to provide enough space for the reaction^30^.

### Preparation and Catalytic Performance of **PCL@MCNC**

The preparation conditions (including precipitation time, enzyme/carrier mass ratio, crosslinker concentration and cross-linking time) of PCL@MCNC were studied.

**Figure 6:**
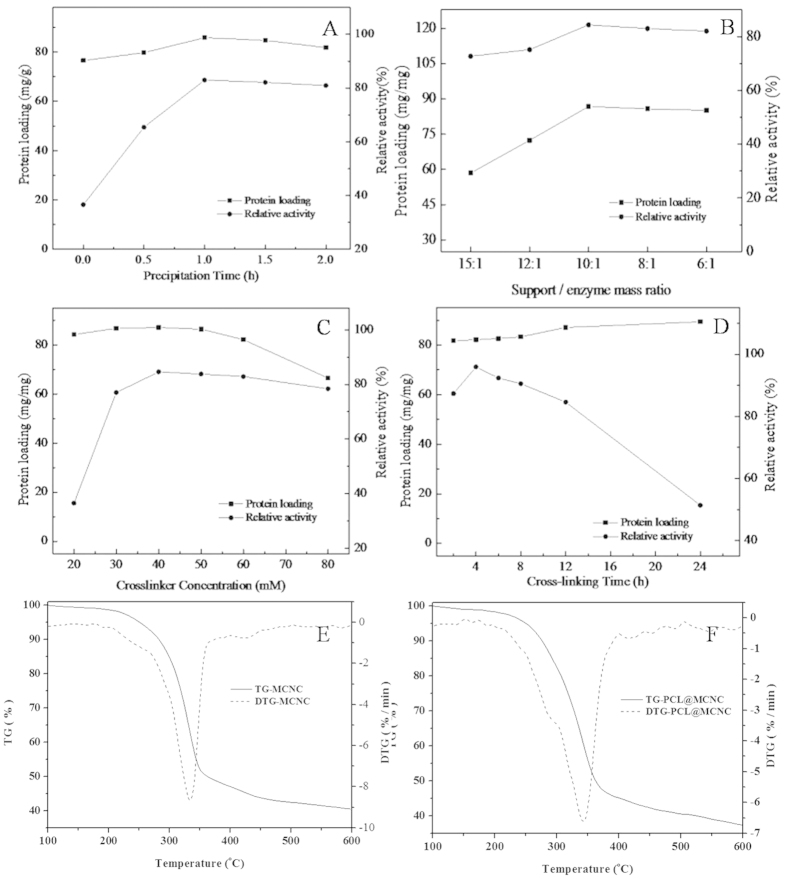
Effects of immobilization conditions on the enzyme loading and activity recovery of PCL@MCNC. Effect of precipitation time (**A**); effect of support/ enzyme mass ratio (**B**); effect of cross-linker concentration (**C**); effect cross-linking time (**D**).

Ammonium sulfate is most commonly used to precipitate the protein^3^,^15^,^31^ and the precipitation time plays an important role in relative activity. Figure 6A shows the effects of precipitation time on the activity recovery of PCL@MCNC. As seen in Fig. 6A, the recovery activity of PCL and protein loading increased initially and then no significant increase was observed for recovery activity or protein amount with longer precipitation time. This could have been due to less zymoprotein precipitated in the short precipitation time causing lower recovery activity. On the other hand, with increased precipitation time, the protein-bound water was stripped and the native structure of the enzyme was disturbed, resulting in decreased enzyme activity. These results were in general agreement with a previous study^15^,^32^. Hence, the most appropriate reaction time was 1 h as this maintained more than 83.0% of activity recovery with an enzyme loading of 85.8 mg enzyme/g MCNC.

As seen in Fig. 6B, with an increase in the support/enzyme mass ratio, the protein loading on MCNC increased and the highest activity recovery (84.4%) was obtained with an enzyme/carrier mass ratio of 10/1. When the support/enzyme mass ratio increased to 15/1, enzyme activity recovery dropped to 72.7%. This was mainly because the appropriate increase in MCNCs support had a positive effect on strengthening the interaction with the enzyme and stabilizing enzyme conformation. Nevertheless, the increase in support may increase the steric hindrance and diffusion resistance of the enzymes, leading to some inhibition in catalyzing the reaction, thus the enzymatic recovery activity decreased gradually^33^.

Figure 6C shows that the glutaraldehyde concentration of 40 mM was most appropriate for cross-linking. According to previous literature, at low glutaraldehyde concentration, the immobilized enzymes were insufficiently cross-linked and released un-bound free enzyme into the aqueous medium. At a high glutaraldehyde concentration, the enzymes were excessively cross-linked, resulting in a decline in enzyme flexibility and inactivation of the enzyme.

A similar trend was observed when the cross-linking time was prolonged (Fig. 6D). It was found that a cross-linking time of 4 h resulted in the highest relative activity with increased coupling time from 2 h to 4 h. Nevertheless, a further increase in cross-linking time led to a decline in enzyme activity due to excess covalent binding between the amino group of MCNCs and the amino group of PCL. For this reason, the most appropriate cross-linking time for the immobilization of PCL was 4 h.

Based on the results shown in Fig. 6, the highest activity of PCL@MCNC was obtained when the precipitation time, support/enzyme mass ratio, cross-linker concentration and cross-linking time were 1 h, 10/1, 40 mM and 4 h, respectively. The relative specific activity was about 95.9% and enzyme loading was as high as 82.2 mg enzyme/g MCNC.

**Figure 7:**
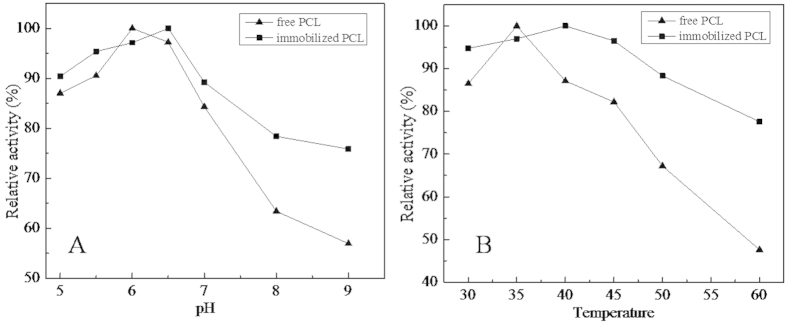
Optimal pH (A) and temperature (B) of PCL@MCNC and native PCL.

In this study, the optimal pH of free PCL and PCL@MCNC was determined in the pH range of 5 to 9. As seen in Fig. 7A, the maximum activities of the free PCL and PCL@MCNC were obtained at pH 6 and 6.5, respectively. The change in the optimal pH for the prepared PCL@MCNC agrees with previous studies which showed similar results after immobilization^34^. The shift in optimal pH value for the PCL@MCNC may have been due to the stronger interactions between the lipase and the carrier material, including hydrogen bonding^35^,^36^,^37^,^38^ as well as electrostatic interactions^39^,^40^. It is noteworthy that the PCL@MCNC showed a higher relative activity compared with free PCL. In particular, at pH 9, the PCL@MCNC retained 75.9% of relative activity, while that of its free counterpart was only 56.8%. In general, PCL immobilized on MCNC carriers exhibited better adaptability to pH, which was similar to the results in a previous report^41^.

The optimal temperature of free PCL and PCL@MCNC was also investigated at the following temperatures (30, 35, 40, 45, 50, 55 and 60 °C) and is shown in Fig. 7B. The maximum relative activity of free PCL was obtained at 35 °C, while PCL@MCNC showed maximum activity at 40 °C and retained more than 88% of itsrelative activity from 30 to 50 °C, whereas free PCL activity was approximately 86.5% and 67.2% at 30 and 50 °C, respectively. When the temperature was increased to 50 °C, the relative activity of free PCL fell sharply and only reached 47.6% of its relative activity at 60 °C. In contrast, PCL@MCNC maintained approximately 77.6% of the relative activity at 60 °C. These results indicated that PCL had better heat resistance after immobilization onto the novel MCNCs.

**Figure 8:**
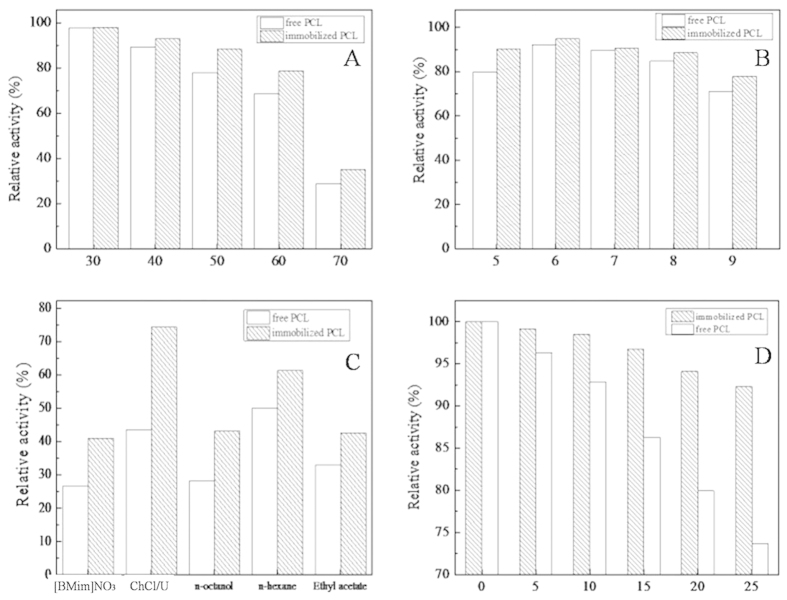
Stability of PCL@MCNC and native PCL. Thermal stability (**A**); pH stability (**B**); solvent tolerance (**C**); storage stability (**D**).

The thermal stability of PCL and PCL@MCNC was determined after incubation at elevated temperatures (30–70 °C). As illustrated in Fig. 8A,PCL@MCNC retained more that 88.4% of its initial activity after 4 h incubation at 50 °C, while that of its free counterpart was only about 77.9%. As seen in Fig. 8A, when the incubation temperature was increased to 70 °C, PCL@MCNC retained about 35.2% residual activity, while the residual activity of free PCL was only 28.8%. The PCL@MCNC was much more stable probably due to the increased rigidity and stability of the secondary structure after immobilization^42^. When comparing the activities of free and immobilized lipase, immobilization resulted in significantly higher thermal stability giving PCL@MCNC an important potential advantage for practical applications in industry.

The pH-stability of PCL@MCNC was investigated at different pH values ranging from 5.0 to 9.0 and the results are shown Fig. 8B. It was apparent that PCL was more active at acidic pH and began to lose activity in alkaline conditions (90.2% vs 77.8%). In accordance with a previous report^43^, enzyme immobilization significantly improved the stability of the enzyme. The PCL@MCNC had much higher pH stability as it maintained more than 77.8% of its initial activity. In contrast, the residual activity of free PCL decreased to around 70.9%. These findings indicated that the conformation of PCL immobilized on the novel MCNCs was more stable, leading to a broad pH tolerance.

As depicted in Fig. 8C, five solvents were used to determine the organic solvent tolerance of both free PCL and PCL@MCNC. PCL@MCNC exhibited better solvent tolerance to these five solvents than its free counterpart. Moreover, *n*-hexane acetate showed the lowest toxicity to free PCL, while deep eutectic solvents (DESs, choline chloride/urea) showed the lowest toxicity to PCL@MCNC as its residual activity was high (74.3%) after 2 h incubation, and was much higher than that of free PCL (43.5%). Similarly, 2 h incubation with *n*-octanol caused nearly 72.9% loss of activity in free PCL, but the PCL@MCNC maintained 43.2% of its initial activity. In comparison with the free counterpart, the PCL-immobilized on MCNCs had higher inactivation resistance to these five solvents. In general, when exposed to organic solvents or ionic liquids, protein-bound water (on the surface of the enzyme) can be easily stripped off and leads to damage of the enzyme’s native structure^30^, which contributes to rapid deactivation of the enzyme. However, after cross-linking with glutaraldehyde on MCNCs, the inner rigidity of the lipase significantly increased and its catalytic conformation was retained. Similar enhanced solvent tolerance as well as maintenance of high catalytic activity in immobilized enzymes have been observed in previous reports^44^.

With the purpose of investigating the storage stability of free and immobilized PCL, both biocatalysts were stored at 4 °C and the stability determined at certain time intervals. As shown in Fig. 8D, compared with free PCL, PCL@MCNC showed superior retention of its initial activity. The PCL@MCNC maintained 92.3% of its initial activity after 25 days of storage, while the corresponding value for PCL was only 73.7%, and this could be explained by modification of the three dimensional structure of the enzyme after immobilization^45^.

A study of Michaelis-Menten kinetics was carried out with various substrate concentrations. The Michaelis-Menten constant (*K*_m_) and V_max_ values for PCL and PCL@MCNC were obtained according to the Hanes-Woolf plots. As seen in Table 1, the apparent K_m_ value for PCL@MCNC was much lower than that for free PCL (12.39 vs 37.90 mM), indicating the greatly enhancing enzyme-substrate affinity after immobilization, which was similar to that reported previously^46^. Also, the V_max_ value for PCL@MCNC was lower than that for PCL (7.39 vs 14.73 mM•min^−1^). Moreover, the V_max_*/K*_*m*_ value for PCL@MCNC was higher than the corresponding value for free PCL (60 × 10^−2^ *vs* 39 × 10^−2^ min^−1^), demonstrating that PCL@MCNC attached to the substrate more easily and had relatively high catalytic efficiency.

### PCL@MCNC-Catalyzed Asymmetric Hydrolysis of KetoprofenEthyl Ester

**Figure 9:**
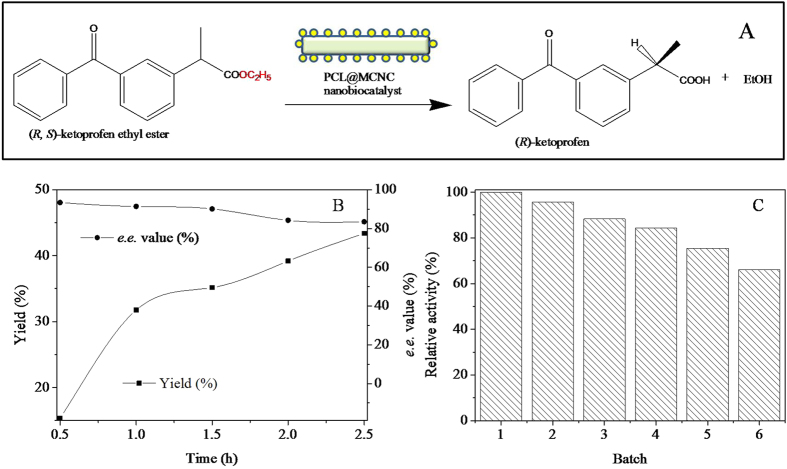
PCL@MNCC-catalyzed asymmetrichydrolysis of ketoprofenethyl ester. Schematic representation of PCL@MNCC-catalyzed asymmetrichydrolysis of ketoprofenethyl ester (**A**); course profile of the enzymatic reaction (**B**); reuse of PCL@MNCC (**C**).

The as-prepared PCL@MCNC was also employed as an efficient nanobiocatalyst for enzymatic asymmetric hydrolysis of ketoprofenethyl ester (Fig. 9A). As depicted in Fig. 9B, the yield of (*R*)-ketoprofen increased markedly with increasing reaction time, and, on the contrast, the product e.e. value decreased gradually. The obtained yield and product e.e. was about 43.4% (a theoretical maximum yield is 50%) and 83.5% at reaction time of 2.5 h, respectively, which was better than the result reported previously^47^. In order to evaluate the operational stability of PCL@MCNC, PCL@MCNC was easily separated from the reaction mixture by an extra magnetic field and reused in a repeated batch process of enzymatic asymmetric hydrolysis of ketoprofenethyl ester. Figure 9C showed that PCL@MCNC could be operated for 6 cycles of successive reuse maintaining more than 66.2% of its original activity, implying that the as-prepared PCL@MCNC had good operational stability. Obviously, the nanobiocatalyst PCL@MCNC indicated great potential for biocatalysis.

## Discussion

The biocompatible MCNCs were prepared and successfully used as an enzyme support for *Pseudomonas cepacia* lipase (PCL) immobilization via the precipitation-cross-linking process. The resulting PCL@MCNCs had improved pH and temperature adaption, higher activity and stronger stability including thermal and storage stability, and enhanced solvent tolerance in comparison with its free counterpart. Also, the kinetics study comparing the immobilized and free enzyme clearly showed that PCL@MCNCs had relatively higher catalytic efficiency than the free counterpart. Furthermore, PCL@MCNC was successfully applied for efficient asymmetric hydrolysis of ketoprofen ethyl ester with high yield and product e.e., and exhibited good operational stability. Obviously, the as-prepared PCL@MCNC is a promising and competitive nanobiocatalyst for biocatalytic reactions.

## Methods

### Preparation of Magnetic Cellulose Nanocrystals (MCNCs)

The preparationof MCNCs was based on the method are ported previously^11^, with some modifications. In a typical experiment, 250 ml of 6 M HCl solution was mixed with 20 g of microcrystalline cellulose and then stirred for 90 min at 90 °C. After continuous hydrolysis, the compound was cooled to stop the reaction and subsequently washed with deionized water for 5 cycles. After centrifugation, the product was filtered and dispersed in a solution containing 50 ml distilled water and 20 ml aqueous solution including 10.72 g of FeCl_2_•4H_2_O and 27.2 g FeCl_3_•6H_2_O. 100 ml acetic acid buffer solution (1%) and chitosan (1.2 g) were added to the suspension with vigorous stirring for 60 min. Then, a suspension of sodium tripolyphosphate (TPP, 1.2 g) and 100 ml NH_4_OH solution were added to the solution with slow stirring for 40 min at 80 °C. Finally, the MCNCs were washed followed by centrifugation five times and then stored in buffer solution.

### Immobilization of Lipase onto MCNCs

Before immobilization, the lipase was purified using the ammonium sulfate deposition method. Typically, 10 g of lipase from *Pseudomonas cepacia*was soaked in 200 ml phosphate buffer solution (PBS, 50 mM, 20% glucose), centrifuged (10,000 rpm, 5 min), and the sediment was discarded. Ammonium sulfate (103.2 g) was slowly added to the clarified supernatant under continuous stirring at 4 °C for 12 h followed by centrifugation at 15000 rpm for 15 min to achieve the green sediment. The resulting lipase was freeze-dried and stored in a refrigerator for further use.

The immobilized PCL on magnetic cellulose nanocrystal (PCL@MCNC) was prepared as follows: typically, 100% saturated ammonium sulfate (80% final saturation) was added drop-wisely into 1 ml PBS (50 mM, pH 7) including free lipase (4.4 mg) and a certain amount of MCNCs. After continuous stirring of the enzyme using a magnetic stirrer for a specified time, the mixture was incubated with a given concentration of 25% glutaraldehyde (GA) for a certain time at 4 °C (220 rpm). Following incubation, the uncross-linked PCL was removed by continuous washing until no protein was detected. The washing solutions were collected to detect the amount of uncross-linked lipase. The amount of immobilized lipase loaded on the MCNCs was calculated as the difference between the initial and the un-crosslinked lipase.

### Characterization of MCNC and PCL@MCNC

The FT-IR analysis was performed using a Tensor 37 spectrometer (Bruker, Germany) equipped with a deuterated triglycine sulfate (DTGS) detector. The FT-IR spectra, acquired at a resolution of 4 cm^–1^ in the range of 400–4000 cm^–1^, were the averages of 64 scans and were recorded against an empty cell as the background. Powder X-Ray diffraction (XRD) was handled with a Bruker D8 Advance X-ray diffractometer with Ni-filtered Cu Ka radiation (k1 = 1.54 Å) generated at a voltage of 40 keV and a current of 40 mA was applied. Magnetism measurements of MCNCs were carried out at RT range from −20000 Oe to 20000 Oe with a vibrating sample magnetometer (VSM) option of the Physical Property Measurement System (PPMS-9, Quantum Design). The morphology of the prepared MCNC and PCL@MCNC was investigated via a Zeiss Merlin SEM (Zeiss, Germany) equipped with an energy dispersive spectrometer (EDS) operated at 10.0 kV. The samples were demagnetized and then sputter-coated with a thin overlayer of gold to prevent sample-charging effects before examination in the microscope. Transmission electron microscopy (TEM) analysis for MCNC and PCL@MCNC was performed with a JEOL JEM-2010 TEM operating at 200kV. A drop (10 μL) of the well-dispersed MCNC suspension was dried on a 300 mesh support film on Double Folding Grids (Beijing Zhongjingkeyi Technology Co., Ltd., China) and analyzed. Thermal gravimetric analysis of MCNC and PCL@MCNC was carried out using a Perkin Elmer Pyris 1 TGA system (Perkin Elmer Inc, USA). The heating rate was 10 °C/min and the heating range was 100–700 °C. The confocal laser scanning microscopy (CLSM) analysis for MCNC and PCL@MCNC was conducted with a TCS SP8 (Leica, Wetzlar, Germany) to determine the fluorescence signal from the FITC (fluorescein *iso*-thiocyanate)-labeled PCL after immobilization onto the MCNC support. The targeted PCL was labeled with FITC as follows: 5 mg of PCL together with 20 mg of FITC were mixed in 1 ml of 5 mM sodium bicarbonate for 24 h at 4 °C, and then the residual FITC was removed from the mixture by the extensive dialysis against distilled water.

### Assay of Enzyme Activity and Protein Concentration

The concentration of protein was measured according to the standard method described by Bradford^48^. Lipase activity was determined by the hydrolysis of p-NPP (4-nitrophenyl palmitate): a given amount of free PCL or PCL@MCNC was dispersed in 0.5 ml PBS solution and then mixed with 0.1 ml of p-NPP solution (150 mM in isopropanol) and incubated for 5 min at 40 °C. Subsequently, 5.3 ml of Na_2_CO_3_ solution (1 M) was added to stop the hydrolysis reaction and the reaction mixture was filtered and detected at 405 nm. One unit of enzyme activity was defined as the amount of enzyme which liberated 1μmol of *p*-nitrophenol/min under the conditions described.

### Determination of Kinetic Parameters and Enzymatic Properties of PCL@MCNC

The enzymatic hydrolysis of p-NPP was used as the model reaction to assay the kinetic parameters of free PCL and PCL@MCNC. The substrate concentrations varied from 20.00 to 150.00 mM in PBS solution (50 mM) at 40 °C. 4.4 μg of enzyme was used each time and the Michaelis-Menten constant (*K*_*m*_) and V_max_ values were calculated from Hanes-Woolf plots.

### Optimal pH and Temperature

The activities of both free and PCL@MCNC were assayed over the pH range from 5 to 9 and the temperature range from 30 to 60 °C, respectively.

### Thermal Stability and pH Stability

Free PCL and PCL@MCNC (27.1 mU) were incubated in PBS (50 mM) at various pH values (5–9, 4 h) and different temperatures (30–70 °C, 2 h). The residual activity was assayed as above.

### Solvent Tolerance

Free PCL and PCL@MCNC (27.1 mU) were incubated in 0.1 ml of various solvents ([BMIM]NO_3_, choline chloride and urea (ChCl:U)-based deep eutectic solvent, *n*-octyl alcohol, *n*-hexane, ethyl acetate) for 2 h. The residual activity of the enzyme was assayed as above.

### Storage Stability

Free PCL and PCL@MCNC (27.1 mU) were stored at 4 °C and stability determined at specified time intervals. The residual activity of the enzyme was assayed as above.

### PCL@MCNC-Catalyzed Asymmetric Hydrolysis of Ketoprofen Ethyl Ester

In a typical experiment, 5 mL PBS (50 mM, pH 6.5) containing Triton X-100 (1.5% wt) and ketoprofen ethyl ester (final concentration 3  mM) added to a 25-ml Erlenmeyer flask capped with a septum. The enzymatic reaction was initiated by 61.5U PCL@MNCC at 35 °C and a stirring rate of 200 r/min. Samples (50 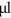) were withdrawn at specified time intervals from the reaction system. The formed product and the residual substrate were extracted with ethyl acetate (2 × 200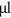 ) prior to HPLC analysis.

### Reusability of **PCL@MCNC**

In order to study the reusability of PCL@MCNC, the PCL@MCNC was readily recycled by magnetic forces at the end of each cycle and then used for the next cycle. In the aqueous monophasic system, the reaction was performed according to above method and repeated over six batches (2.5 h per batched). Between batches, the PCL@MCNC were recovered by magnetic forces and washed twice with distilled water. Then the PCL@MCNC was suspended again in a fresh batch of reaction medium. The relative activity of the PCL@MCNC employed for the first batch was defined as 100%.

### HPLC Analysis

The samples were analyzed with an Agilent 1100 HPLC using aCHIRALPAK OJ-H column (4.6 × 250 mm, Daicel, Japan) and UV detection at 254 nm.The mobile phase was a mixture of *n*-hexane, isopropanol and acetic acid (90/10/0.5, v/v) at a flow rate of 1.0 mL/min. The retention times of ketoprofen ethyl ester, (*R*)-ketoprofen and (*S*)-ketoprofen were 8.4, 11.3 and 14.0 min, respectively. All data reported are averages of experiments performed at least three times, with less than 2.0% standard deviation.

## Additional Information

**How to cite this article**: Cao, S.-L. *et al.* Preparation and Characterization of Immobilized Lipase from Pseudomonas Cepacia onto Magnetic Cellulose Nanocrystals. *Sci. Rep.* **6**, 20420; doi: 10.1038/srep20420 (2016).

## Figures and Tables

**Table 1:** Apparent kinetics parameters of free PCL and PCL@MCNC.

Enzyme preparation	K_m_(mM)	V_max_ (mM.min^−1^)	V_max_/K_m_ (min^−1^)
Free enzyme	37.90	14.73	0.39
PCL@MCNC	12.39	7.39	0.60
